# Immediate Beneficial Effects of Mental Rotation Using Foot Stimuli on Upright Postural Stability in Healthy Participants

**DOI:** 10.1155/2013/890962

**Published:** 2013-12-26

**Authors:** Tsubasa Kawasaki, Takahiro Higuchi

**Affiliations:** Department of Health Promotion Science, Graduate School of Human Health Science, Tokyo Metropolitan University, 1-1 Minami-Osawa, Hachioji, Tokyo 192-0397, Japan

## Abstract

The present study was designed to investigate whether an intervention during which participants were involved in mental rotation (MR) of a foot stimulus would have immediate beneficial effects on postural stability (Experiment 1) and to confirm whether it was the involvement of MR of the foot, rather than simply viewing foot stimuli, that could improve postural stability (Experiment 2). Two different groups of participants (*n* = 16 in each group) performed MR intervention of foot stimuli in each of the two experiments. Pre- and postmeasurements of postural stability during unipedal and bipedal standing were made using a force plate for the intervention. Consistently, postural sway values for unipedal standing, but not for bipedal standing, were decreased immediately after the MR intervention using the foot stimuli. Such beneficial effects were not observed after the MR intervention using car stimuli (Experiment 1) or when participants observed the same foot stimuli during a simple reaction task (Experiment 2). These findings suggest that the MR intervention using the foot stimuli could contribute to improving postural stability, at least when it was measured immediately after the intervention, under a challenging standing condition (i.e., unipedal standing).

## 1. Introduction

A mental rotation (MR) task using a visual stimulus of a pictured body part, typically a hand or foot, asks participants to judge whether the stimulus is the right or left hand/foot (i.e., laterality judgment). The time required for judging (i.e., reaction time) increased as the linear function of angle rotation [1, 2]. Even if a stimulus was presented with no rotation, the reaction time was delayed when participants kept their right hand behind their back so that the orientation of the hand was far from that of the stimulus [3]. The reaction time was nearly equivalent to the time of the actual body movement to the orientation of the stimulus; for example, the reaction time at 90 degrees is similar to the time it would actually take to move the hand 90 degrees [2]. Moreover, neuroimaging studies showed that the brain regions in the posterior parietal cortex and the precentral cortex, which are involved in motor planning [4, 5], were activated while performing the MR of body parts [6, 7]. Based on these findings, it has been generally considered that MR of a body part involves cognitive processes used for both motor imagery and motor execution [1, 2, 8].

The present study was designed to investigate with two experiments whether the intervention during which participants were involved in MR of the foot would have immediate beneficial effects on postural stability. If MR of a body part involves cognitive processes used for both motor imagery and motor execution, then repeated MR for a certain period of time might activate such cognitive processes and, as a result, contribute to improving motor performance. In fact, our previous study showed that interventions that involved participants in motor imagery [9] and motor execution [10] of a body part had immediate beneficial effects on postural stability during upright unipedal standing [9, 10]. We investigated whether a similar effect would be observed when MR, instead of motor imagery, was used for intervention.

In the present study, a foot stimulus was selected as the body part to be used in MR based on the foot's essential role in controlling upright human posture [11–13]. The first experiment was designed to examine the effect of the MR intervention using the foot stimuli as compared with that using a car (i.e., no body-related stimuli). The second experiment was designed to investigate whether the involvement of MR of the foot, rather than simply viewing foot stimuli, could improve postural stability.

## 2. Experiment 1

### 2.1. Method

#### 2.1.1. Participants

Sixteen young adults participated (nine women and seven men, age 22.8 ± 4.2 years). Inclusion criteria were (a) no visual disability, (b) no sensory or motor impairments that could influence their balance, and (c) the ability to maintain balance with unipedal standing for more than 60 seconds. All participants had a right dominant foot. All participants gave informed consent prior to participating in the study. Experimental protocols were approved by the Institutional Ethics Committee of Tokyo Metropolitan University (approval number 24–42). The tenets of the Declaration of Helsinki were followed.

#### 2.1.2. Apparatus and Materials

**Figure 1:**
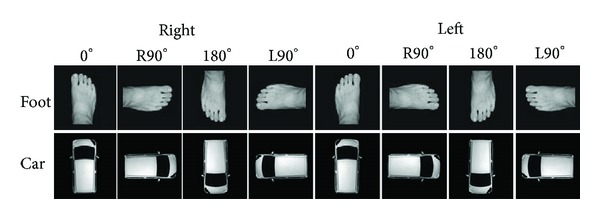
Schematic representation of three visual stimuli (foot and car) at four angles.

A personal computer (VGN-SR72B, Sony Corp., Japan) and presentation software (EXPLAB for Windows, ver. 1.3, Yachiyo Shuppan, Japan) were used for the MR intervention to present MR stimuli and collect reaction times. Two digital images (foot and car: approximately 9.5 cm in height and 4.5 cm in width) presented at four angles, 0°, right 90° (R90°), 180°, and left 90° (L90°), were used for the MR stimuli (Figure 1). A force plate (type 9286AA: Kistler Instrumente AG, Winterthur, Switzerland) was used to determine the center of pressure during unipedal and bipedal standings.

#### 2.1.3. Task and Dependent Variables

*Mental Rotation Task for Intervention.* The participants sat comfortably in front of a computer screen. The screen was positioned at a 50 cm distance from the participants' eyes. Their hands were occluded with a cloth. The stimulus appeared on the center of the screen. For the foot stimuli, the participants were asked to determine whether the stimulus was the left or right foot as quickly as possible and indicate it by pressing a predetermined key (J for right and F the left) with the predetermined finger (the right or left index finger, resp.). For the car stimuli, they were asked to determine which side of the headlights was painted black as quickly as possible and indicate it in the same manner as for the foot stimuli. For each of the two stimuli (i.e., the foot and the car), participants performed a total of 160 main trials (20 trials × four angles × laterality [right and left]). Prior to the main trials, they performed 24 practice trials (three trials × four angles × laterality) to familiarize themselves with the MR task. Performing the MR task for each stimulus took approximately 10 minutes. The dependent variable was the reaction time. Based on previous studies [1–3, 8, 14], reaction times slower than 3,500 ms were excluded from the analysis.

*Postural Stability Task*. Stability of upright posture (body sway) was evaluated while the participants stood barefoot and tried to remain as still as possible during unipedal and bipedal standing on a force plate. While standing, their eyes were directed to an eye-level fixation point at a distance of 180 cm ahead. For the unipedal standing, participants stood on their nondominant (left) leg with their arms folded across their chest and flexed their right knee to approximately 90°. For bipedal standing, they stood with their feet close together and their arms along the sides of their body.

To calculate the postural sway values, the center-of-pressure data, collected at the 50 Hz sampling frequency, were obtained from the force plate. The data were low-pass-filtered at 6 Hz, since most of the power of the signal was <2 Hz [15]. The participants performed three 60-second trials under each standing condition with a one-minute interval between trials. The order of the standing posture was counterbalanced among the participants.

The postural sway values were expressed as the mean velocity of sway (MV), the mean velocity of sway in the anterior-posterior directions (A-P velocity) and the medial-lateral directions (M-L velocity), and the root mean square area (RMS area). Each of the four values was calculated using the following formulas:

(1) mean  velocity  of  sway  =1S∑i=1n(AXi−AXi)2+(AYi−AYi)2,mean  velocity  of  sway  in  the  A-P  direction  =1S∑i=1n(AXi−AXi)2,mean  velocity  of  sway  in  the  M-L  direction  =1S∑i=1n(AYi−AYi)2,root  mean  square  area  =π(1n∑i=1n(Xi−Xm)2+(Yi−Ym)2)2.

**Figure 2:**
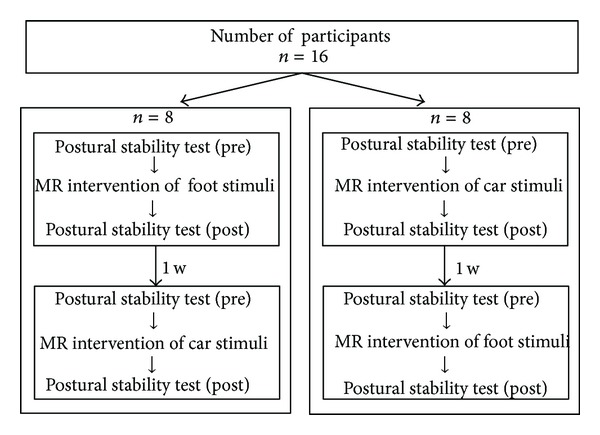
Schematic figure of experimental protocol in Experiment 1. All participants were involved in two intervention sessions. The order of the stimuli for mental rotation (i.e., the foot or car) presented on the first intervention day was counterbalanced on the second.

*Protocols*. We used a randomized crossover design. The experiment consisted of two-day sessions at least one week apart. The order of the intervention stimuli presented on the first day is shown in Figure 2. On each day, we examined the immediate effect of the MR using either foot or car stimuli on postural stability. Measurements of postural stability during unipedal and bipedal standing were made using the force plate before and immediately after the 10-minute MR intervention.

#### 2.1.4. Data Analysis

As a preliminary analysis of the MR intervention, the reaction times were analyzed with a stimulus (foot, car) × stimulus angle (0°, R90°, 180°, L90°) analysis of variance (ANOVA) with repeated measures of both factors. This preliminary analysis was necessary for determining whether the participants were definitely involved in the MR of the foot. That is, if they were involved in the MR, then the reaction times should increase as the angle of rotation became larger [1–3, 8, 16–18].

For each dependent variable of postural control, a statistical test was conducted separately for bipedal and unipedal standing tasks. Individual sway values were analyzed with an intervention (foot, car) × session (pre, post) ANOVA with repeated measures on both factors. The level of significance was set at *P* < 0.05.

### 2.2. Results and Discussion

**Figure 3:**
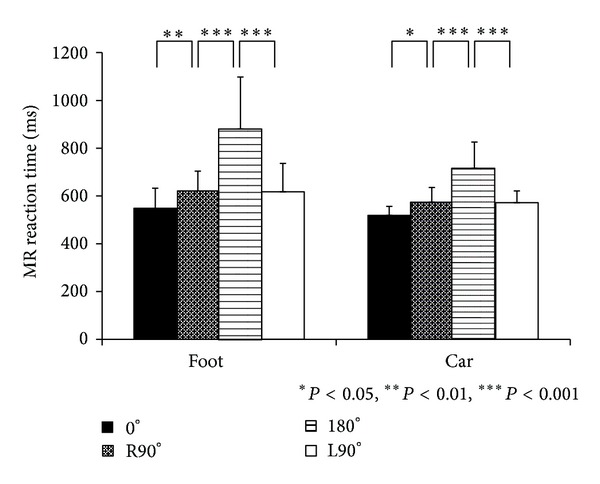
Mean reaction time at the different MRs for foot and car stimulus angles in Experiment 1. Error bars depict the standard deviation of the mean.

The mean reaction times for each stimulus are shown in Figure 3. The main effect of the stimulus angle was significant (*F* (3, 45) = 80.24, *P* < 0.001). Post hoc analyses showed that the reaction time was significantly longer with increasing rotation angle (0° versus R90°: *t* (45) = 3.24, *P* = 0.002; R90° versus 180°: *t* (45) = 14.47, *P* < 0.001; 180° versus L90°: *t* (45) = 11.14, *P* < 0.001; 0° versus L90°: *t* (45) = 3.33, *P* = 0.002). This suggests that the participants were definitely involved in the MR of the foot. There was also an interaction between the stimulus and the stimulus angle for the reaction time (*F* (3, 45) = 12.67, *P* < 0.001, Figure 3).

For postural stability during unipedal standing, the main effect of the session was significant on the MV, A-P velocity, and M-L velocity (*F* (1, 15) = 21.79, *P* < 0.001; *F* (1, 15) = 14.84, *P* = 0.002; and *F* (1, 15) = 20.74, *P* < 0.0001, resp., Table 1). These velocities were significantly lower immediately after the MR intervention. There was a significant interaction between the intervention and the session on the MV and A-P velocity (*F* (1, 15) = 4.56, *P* = 0.04 and *F* (1, 15) = 4.97, *P* = 0.04, resp.) but not on the M-L velocity or the RMS area. Post hoc analyses showed that, when the foot stimuli were used, the MV and A-P velocity were significantly slower after the MR intervention than before the intervention (*F* (1, 30) = 23.42, *P* < 0.001 and *F* (1, 30) = 18.50, *P* < 0.001, resp.). When measured immediately after the MR using the car stimuli, no significant differences between the pre- and postsession were obtained for any of the sway values. For postural stability during bipedal standing, there were no significant differences for any of the sway values immediately after performing MR using both stimuli.

These findings suggest that MR intervention using foot stimuli would significantly improve postural stability, such as body velocity, immediately after the intervention. However, this was likely to be the case only when individuals tried to control their posture under a more challenging condition, such as unipedal standing.

## 3. Experiment 2

Experiment 1 showed the beneficial effects of MR intervention using the foot stimuli on postural stability. To investigate whether such beneficial effects truly resulted from the MR, Experiment 2 was designed to compare the effect of the intervention using MR of the foot stimuli with that using the simple reaction (SR) to the foot stimuli (i.e., MR was not requested). We also compared the effects of these interventions with that of no intervention (i.e., a resting period was inserted between the pre- and postmeasurement of postural stability) to eliminate the possibility that an improvement in the postmeasurement was not derived simply from a repetition of the measurements.

### 3.1. Method

#### 3.1.1. Participants

Sixteen young adults participated (ten women and six men, age 22.5 ± 3.9 years). Inclusion criteria were the same as those in Experiment 1. Two participants had taken part in Experiment 1. Because more than two months had passed since they participated in Experiment 1, we believe that their experience in Experiment 1 would not have been carried over to this experiment. The experiment's protocols were approved by the Institutional Ethics Committee of the Tokyo Metropolitan University (approval number 24–42). Each participant gave written informed consent prior to participating.

#### 3.1.2. Protocols and Data Analyses

The apparatus and protocols were generally the same as those in the first experiment, except that participants performed three kinds of intervention (MR, SR, and resting). The experiment consisted of three-day sessions with at least one week between each session. We examined the immediate effect of each intervention on each day.

The protocol of the MR intervention was the same as that in Experiment 1, except that only foot stimuli were used for the MR and SR tasks. A foot stimulus identical to that used in Experiment 1 was presented on the computer screen at one of four rotation angles. For the SR intervention, the foot stimuli were presented with a blocked design; that is, either the right or the left foot stimuli were presented consecutively. This block design was helpful for letting the participants understand that, in the SR intervention, they were not asked to give a laterality judgment but to respond as quickly as possible. In the SR intervention, regardless of the rotation angle of the stimulus, participants tried to press the predetermined key (J for the right and F for the left) with the predetermined finger (right or left index finger, resp.) as quickly as possible. The participants performed 240 trials as main trials (i.e., 30 trials × four angles × laterality). The main trials were divided into two blocks; in each of the blocks, only the right or left foot was presented. A rest period of two minutes was inserted between the blocks. Whichever side of the foot was presented in the first block was counterbalanced in the second. Prior to performing the main trials, participants performed 24 trials (i.e., three trials × four angles × laterality). The SR intervention took approximately 10 minutes. For the resting intervention, participants were asked to sit in a chair for 10 minutes between the pre- and postmeasurements of postural sway.

Dependent variables and data analyses were identical to those in Experiment 1. The reaction times for the MR and SR were analyzed separately with a stimulus angle (0°, R90°, 180°, L90°) ANOVA. For each dependent variable of postural control, a statistical test was conducted separately for unipedal and bipedal standing tasks. Individual sway values were analyzed with an intervention (MR, SR, resting) × session (pre, post) ANOVA with repeated measures of both factors.

### 3.2. Results and Discussion

**Figure 4:**
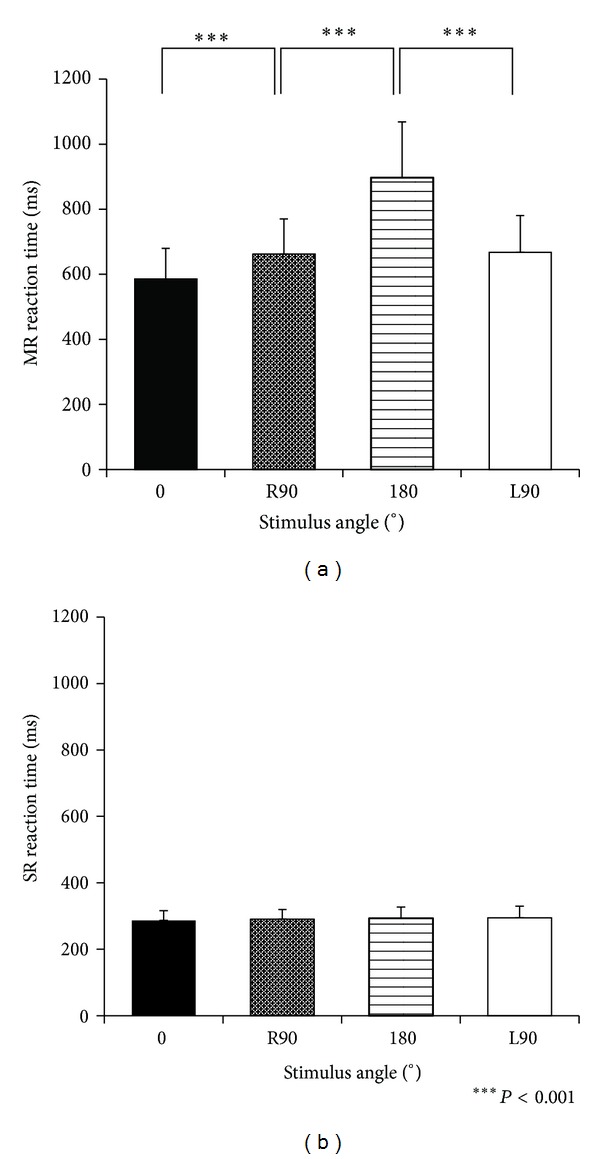
Mean reaction times for (a) MR of foot stimulus angle and (b) SR of foot stimulus angle in Experiment 2. Error bars depict the standard deviation of the mean.

The mean reaction times for each stimulus are shown in Figure 4. For the MR reaction times, the significant main effect of the stimulus angle was significant (*F* (3, 45) = 80.84, *P* < 0.001). Post hoc analyses showed that reaction time significantly increased with increasing stimulus angle (0° versus R90°: *t* (45) = 3.63, *P* = 0.01; R90° versus 180°: *t* (45) = 11.09, *P* < 0.001; 180° versus L90°: *t* (45) = 10.84, *P* < 0.001; 0° versus L90°: *t* (45) = 3.88, *P* < 0.001). There was no significant difference in SR reaction time for the foot stimuli. This suggests that participants were involved in the MR of the foot while performing the MR task but not during the SR task.

For postural stability during unipedal standing, the main effect of the session was significant on the MV, A-P velocity, and M-L velocity (*F* (1, 15) = 19.77, *P* < 0.001; *F* (1, 15) = 13.46, *P* = 0.002; and *F* (1, 15) = 14.15, *P* = 0.002, resp., Table 2) but not on the RMS area. The three velocities were significantly lower immediately after the intervention. There were significant interactions between the intervention and session on three velocities (*F* (2, 30) = 7.29, *P* = 0.003; *F*  (2, 30) = 8.66, *P* = 0.001; and *F*(2, 30) = 3.60, *P* = 0.04, resp., Table 2) but not on the RMS area. Post hoc analyses showed that, when measured immediately after the MR intervention, the MV, A-P velocity, and M-L velocity were significantly lower (*F* (1, 45) = 33.72, *P* < 0.001; *F* (1, 45) = 30.50, *P* < 0.001; and *F* (1, 45) = 20.74, *P* < 0.001, resp., Table 2). No such difference was shown immediately after the SR intervention or resting intervention for any of the sway values. For postural stability during bipedal standing, there was not any significant difference or any of the sway values immediately after performing any intervention.

The results replicated the findings of Experiment 1 that the MR intervention using foot stimuli was likely to have a beneficial effect for postural control only under a challenging condition, such as unipedal standing, which confirmed the reliability of the effect of the MR intervention.

## 4. General Discussion

The results obtained from the two experiments have generally shown that the MR intervention using the foot stimuli was likely to have immediate beneficial effects on postural stability only in a unipedal standing condition. During that condition, the magnitude of the three velocity variables regarding postural sway, but not the RMS area, decreased significantly after the intervention. In fact, these findings were consistent with the previous study demonstrating that, only during unipedal standing condition, individuals who were involved in motor imagery [9] and motor execution [10] of a body part and of the whole body had immediate beneficial effects on postural stability. Therefore, it seems likely that intervention involving MR of the foot stimuli could contribute to improving the postural stability measured immediately after intervention under a challenging standing condition (i.e., unipedal standing).

Throughout the two experiments, the effects of the MR intervention on postural stability during unipedal standing were observed with some of the three velocity variables but not with the RMS area measurement. A previous study reported that patients with proprioceptive disorders showed an increase in length as compared to sway area, whereas patients with labyrinthine disorder showed an increase in sway area as compared to length [19]. From these findings, it was proposed that the measurement of length and velocity might represent a proprioceptive function, whereas the sway area (i.e., the RMS area) might represent a vestibular variable function. Considering that the ability to quickly perform MR is strongly related to proprioceptive function [3, 17, 18, 20], the MR intervention, during which participants were involved in motor imagery and motor execution of a body part, could contribute to improving postural stability by improving the proprioceptive sensitivity to detect postural perturbations.

For bipedal standing, no beneficial effects of any interventions were found. The lack of beneficial effects could be related to the facts that (a) the subcortical areas (e.g., the basal ganglia and cerebellum) are more likely to be involved in bipedal standing than is the cerebral cortex [21, 22] and (b) postural control for bipedal standing is a stable condition and a familiar action pattern experienced in daily life [12]. Therefore, because bipedal standing is automatically controlled as compared to unipedal standing, MR of the body parts involved in the cerebral cortex might not effectively influence the performance of bipedal standing.

In conclusion, the present study showed that MR intervention using foot stimuli is likely to have some beneficial effects for the improvement of postural stability during unipedal standing but not during bipedal standing, which would indicate that the MR intervention using foot stimuli is beneficial only for more challenging postural standings, such as unipedal standing. Future studies are required to investigate carryover effects on postural stability via MR intervention using foot stimuli to determine whether it can be used effectively in a clinical setting. MR intervention could potentially be used in clinical setting for individuals who do not have any cognitive disorder, such as fragile elderly people or patients with lower-extremity orthopedic problems. It is important to note that some patients with central nerve system diseases (e.g., stroke, Parkinson's disease, or dystonia) have difficulty performing MR [23–25]. Therefore, in these patients, MR intervention should be used with care.

## Figures and Tables

**Table 1:** Comparison of pre- and postintervention in each sway value for two standings in the two kinds of interventions in Experiment  1.

	Unipedal standing			Bipedal standing		
	Pre	Post		Pre	Post	
MR of foot stimuli						
Mean velocity (cm/sec)	3.42 ± 0.78	3.07 ± 0.78	∗∗∗	1.15 ± 0.25	1.13 ± 0.26	n.s
A-P velocity (cm/sec)	2.07 ± 0.52	1.81 ± 0.59	∗∗∗	0.64 ± 0.13	0.62 ± 0.16	n.s
M-L velocity (cm/sec)	2.43 ± 0.57	2.04 ± 0.57	n.s	0.84 ± 0.18	0.85 ± 0.20	n.s
RMS area (cm^2^)	3.12 ± 1.08	3.04 ± 1.18	n.s	2.04 ± 1.11	2.04 ± 1.01	n.s
MR of car stimuli						
Mean velocity (cm/sec)	3.31 ± 0.82	3.18 ± 0.71	n.s	1.17 ± 0.28	1.10 ± 0.26	n.s
A-P velocity (cm/sec)	1.94 ± 0.43	1.87 ± 0.50	n.s	0.63 ± 0.12	0.62 ± 0.15	n.s
M-L velocity (cm/sec)	2.36 ± 0.44	2.14 ± 0.46	n.s	0.86 ± 0.19	0.83 ± 0.20	n.s
RMS area (cm^2^)	3.10 ± 1.05	3.20 ± 1.01	n.s	2.05 ± 1.09	1.90 ± 1.00	n.s

***P < 0.001.

**Table 2:** Comparison of pre- and postintervention in each sway value for two standings in the three kinds of interventions in Experiment  2.

	Unipedal standing			Bipedal standing		
	Pre	Post		Pre	Post	
MR of foot stimuli						
Mean velocity (cm/sec)	3.50 ± 1.03	3.08 ± 0.88	∗∗∗	1.21 ± 0.37	1.18 ± 0.41	n.s
A-P velocity (cm/sec)	2.30 ± 0.77	2.01 ± 0.71	∗∗∗	0.75 ± 0.20	0.73 ± 0.23	n.s
M-L velocity (cm/sec)	2.17 ± 0.62	1.92 ± 0.48	∗∗∗	0.79 ± 0.32	0.76 ± 0.34	n.s
RMS area (cm^2^)	2.95 ± 1.49	2.82 ± 1.20	n.s	1.75 ± 1.00	1.95 ± 1.31	n.s
SR of foot stimuli						
Mean velocity (cm/sec)	3.40 ± 0.76	3.26 ± 0.73	n.s	1.17 ± 0.22	1.17 ± 0.31	n.s
A-P velocity (cm/sec)	2.20 ± 0.59	2.13 ± 0.56	n.s	0.73 ± 0.17	0.75 ± 0.24	n.s
M-L velocity (cm/sec)	2.13 ± 0.51	2.05 ± 0.46	n.s	0.74 ± 0.20	0.73 ± 0.22	n.s
RMS area (cm^2^)	2.78 ± 0.91	2.83 ± 1.06	n.s	1.93 ± 0.98	2.29 ± 1.63	n.s
Rest						
Mean velocity (cm/sec)	3.32 ± 0.76	3.22 ± 0.75	n.s	1.21 ± 0.31	1.14 ± 0.33	n.s
A-P velocity (cm/sec)	2.14 ± 0.59	2.09 ± 0.61	n.s	0.77 ± 0.22	0.73 ± 0.23	n.s
M-L velocity (cm/sec)	2.07 ± 0.54	1.97 ± 0.43	n.s	0.77 ± 0.23	0.71 ± 0.23	n.s
RMS area (cm^2^)	2.69 ± 0.82	3.04 ± 0.89	n.s	2.04 ± 2.15	2.06 ± 2.44	n.s

***P < 0.001.

## Abbreviations

MR: Mental rotation
COP: Center of pressure
MV: Mean velocity of sway
A-P velocity: Mean velocity of sway in the anterior-posterior directions
M-L velocity: Mean velocity of sway in the medial-lateral directions
RMS area: Root mean square area
SR: Simple reaction.
